# Genomic Analysis of the Chicken Infectious Anemia Virus in a Specific Pathogen-Free Chicken Population in China

**DOI:** 10.1155/2016/4275718

**Published:** 2016-05-19

**Authors:** Yang Li, Yixin Wang, Lichun Fang, Jiayuan Fu, Shuai Cui, Yingjie Zhao, Zhizhong Cui, Shuang Chang, Peng Zhao

**Affiliations:** College of Veterinary Medicine, Shandong Agricultural University, Tai'an 271018, China

## Abstract

The antibody to chicken infectious anemia virus (CIAV) was positive in a specific pathogen-free (SPF) chicken population by ELISA test in our previous inspection, indicating a possible infection with CIAV. In this study, blood samples collected from the SPF chickens were used to isolate CIAV by inoculating into MSB1 cells and PCR amplification. A CIAV strain (SD1403) was isolated and successfully identified. Three overlapping genomic fragments were obtained by PCR amplification and sequencing. The full genome sequence of the SD1403 strain was obtained by aligning the sequences. The genome of the SD1403 strain was 2293 bp with a nucleotide identity of 94.8% to 98.5% when compared with 30 referred CIAV strains. The viral proteins VP2 and VP3 were highly conserved, but VP1 was not relatively conserved. Both amino acids 139 and 144 of VP1 were glutamine, which was in accord with the low pathogenic characteristics. In this study, we first reported that CIAV exists in Chinese SPF chicken populations and may be an important reason why attenuated vaccine can be contaminated with CIAV.

## 1. Introduction

Chicken infectious anemia virus (CIAV) can cause the atrophy of bone marrow hematopoietic and lymphoid tissues (e.g., thymus) in young chickens, leading to aplastic anemia and immune suppression. The infection has been very common in chicken populations worldwide [1–4] since first reported CIAV in 1979 [5].

CIAV belongs to the* Gyrovirus* genus of the Circoviridae family, and the genome has three open reading frames (ORFs) which encode VP1, VP2, and VP3 [2]. VP1 and VP2 are protective antigen proteins which are encoded to produce neutralizing antibodies [6]. Except for VP1, the amino acid (AA) composition of CIAV was relatively conserved. Previous studies have indicated that AAs 139–151 are a highly variable region of the VP1 gene, and AA 139 or 144 could especially affect virus replication and cell infection [7]. Generally speaking, if both AA 139 and 144 in the loci are glutamines, replication and infective ability of the virus are relatively weaker [7].

It was reported that CIAV often contaminated attenuated vaccines to infect chicken populations [4, 8], and the use of CIAV-contaminated specific pathogen-free (SPF) chicken embryos is one of the important reasons for CIAV infection of avian attenuated vaccine. In the current study, a CIAV strain was isolated from a Chinese SPF chicken population, and the sequence of its full genome was analyzed.

## 2. Materials and Methods

### 2.1. Background of the SPF Chicken Population

According to guidelines, the detection of antibodies in SPF chicken flocks must be carried out regularly to evaluate the infection status of different pathogenic bacteria and viruses. CIAV antibody test kit (IDEXX, Beijing, China) was used to investigate the positive rate of CIAV antibody in one of SPF flocks in China. The positive rate of CIAV antibody in this flock was 20%. Blood samples were collected from those chickens, and blood DNA was isolated using a DNA extraction kit (Omega, USA). Subsequently, PCR experiments were carried out according to the methods in previous studies to detect viral genome DNA of CIAV [8].

### 2.2. Isolation and Detection of Virus

Blood samples positive for CIAV antibody were collected from ten individual chickens and then inoculated into MDCC-MSB1 cells. This cell line was purchased from ATCC and passaged in our lab for fifteen generations. The blind passage was carried out every three days, until the emergence of cytopathogenic effects (CPE). After CPE, the media of MSB1 cells were collected and repeatedly frozen and thawed three times. The media were then centrifuged, and DNA was extracted using a DNA extraction kit (Tiangen, Beijing, China) from the supernatant. PCR experiments for identification were carried out according to previous studies [8].

### 2.3. Whole Genome Sequencing

According to published CIAV genome sequences (Table 1), three pairs of primers were designed (Primer Premier 5.0) and three overlapping fragments were amplified, respectively (Table 2). Those fragments were subcloned into PMD-18T vectors (Takara, Dalian, China) and sequenced by Sanger method (Sangon, Shanghai, China). Then, the whole genome sequences of the isolated strains were obtained by assembling those overlapping fragments using Lasergene 7.1 software.

### 2.4. Sequence Analysis of Isolated Strain

In order to compare the homology of CIAV with different reference strains, the homology of the whole genome and its coding regions (VP1, VP2, and VP3) were analyzed by DNAstar software. Phylogenetic trees were reconstructed on the basis of sequences from the whole genome, VP1, VP2, and VP3 respectively, using MEGA5.1 (NJ) software. The mutation sites of AA from the 139 to 151 region of the VP1 loci in different strains were analyzed statistically.

*Nucleotide Sequence Accession Numbers*. The GenBank accession number for the sequences of SD1403 was KU221054.

## 3. Results

### 3.1. The Isolation and Identification of the Virus

**Figure 1:**
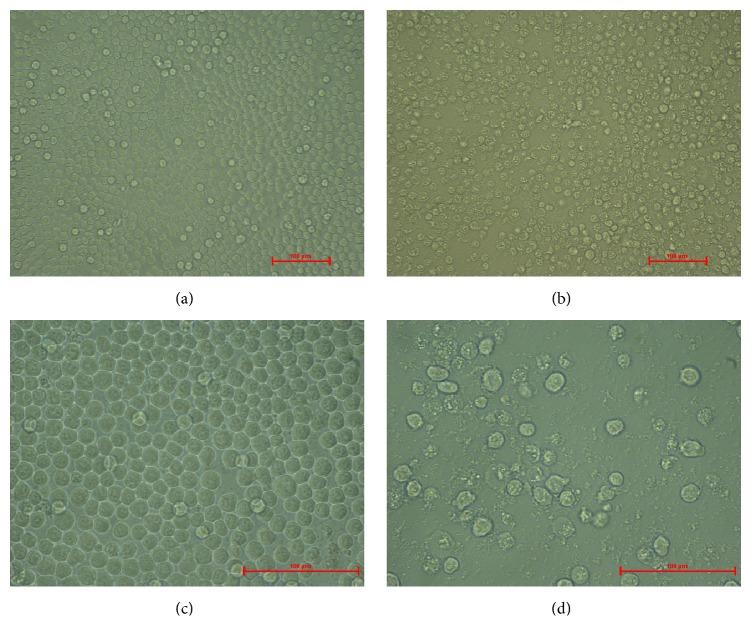
Cytopathic effect of CIAV isolate on MSB1 cells. (a) Normal MSB1 cells (200x). (b) CPE of MSB1 cells infected with CIAV (200x). (c) Normal MSB1 cells (400x). (d) CPE of MSB1 cells infected with CIAV (400x).

**Figure 2:**
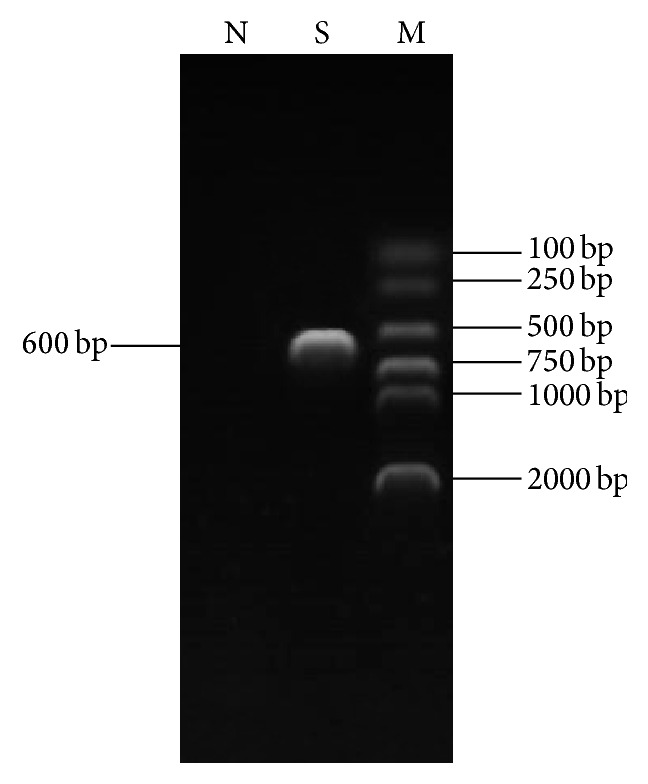
Identification by PCR amplification of the CIAV isolates. N: MSB1 cell without CIAV infection as negative control; S: sample detected in the current study; M: marker.

10^6^ MDCC-MSB1 cells cultured in six-well plates were inoculated with 100 *μ*L blood from ten individual SPF chickens which might be infected with CIAV, and, after four blind passages, the cells infected with one blood sample displayed CPEs. Some cells became round and enlarged, and lysis or disintegrating of cells could be observed, with the number of viable cells decreasing, while the morphology of uninoculated MSB1 cells remained normal (Figure 1). A specific PCR band of about 600 bp was amplified from the DNA of MSB1 cells showing CPE (Figure 2). The result indicated that a CIAV strain was isolated successfully and named as SD1403.

### 3.2. Genome Sequencing of the Isolate

**Figure 3:**
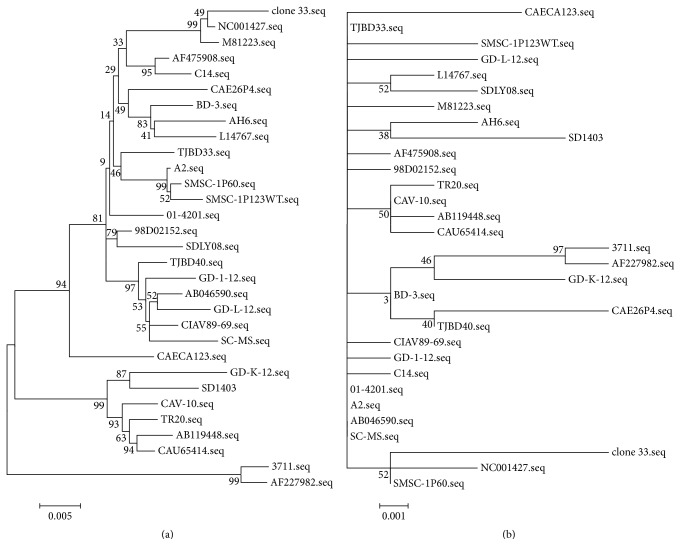
Phylogenetic analysis of different CIAV isolates based on whole genome sequence and VP3 protein. (a) Phylogenetic analysis of CIAV isolates based on whole genome. (b) Phylogenetic analysis of VP3 protein from different CIAV isolates.

The full genome of SD1403 strain was 2298 bp, including three ORFs (VP1, VP2, and VP3). VP1, VP2, and VP3 were 1350 bp, 651 bp, and 366 bp in length, respectively. Comparison between the whole genome sequences of the SD1403 strain and 30 other strains was carried out, and the results demonstrated that the homology was 94.8%–98.5%. A phylogenic tree was constructed using whole genome sequence of SD1403 showing that SD1403 was closely related to the GD-K-12 strain, which has low pathogenicity and was isolated in China in 2013 (Figure 3(a)).

Compared with other reference strains, the homology of VP1, VP2, and VP3 from the SD1403 strain was 97.7%–98.3%, 98.3%–99.2%, and 97.8%–99.5%, respectively. It was shown that the sequences of the three genes were highly conserved. VP2 was more conserved than VP1, with the most conserved gene being VP3. The phylogenetic trees were constructed on the basis of VP1, VP2, and VP3 proteins. Phylogenetic tree which was constructed on the basis of VP1 protein was similar to that constructed on the basis of whole genome. It showed that SD1403 and GD-K-12 were in the same clade, indicating that VP1 was more representative between the strains. The SD1403 and GD-K-12 strains were isolated in China and had common characteristics, with AA 139 and 144 of VP1 being glutamine (Table 1). Phylogenetic tree constructed on the basis of VP2 protein was similar to that constructed on the basis of VP3 protein, indicating that both of them were relatively conserved (Figure 3(b)).

## 4. Discussion

A lot of attenuated vaccines for poultry have been confirmed to contain some exogenous viruses. The most reported viruses are Avian leukosis virus (ALV) [9, 10] and Reticuloendotheliosis virus (REV) [11–13], with CIAV also being reported [8]. The main reason for this phenomenon is that vaccines made from SPF chicken embryos contaminated by viruses have been used. These viruses (ALV, CIAV, REV, etc.) are mainly transmitted vertically. In China, it has been previously reported that SPF chicken populations were infected with ALV [14]. This is the first report of a CIAV strain isolated and identified in a SPF chicken population in China.

To analyze the genetic variation of this strain and track its possible origin, we sequenced its whole genome. Similar to other CIAV strains, the genomic sequence of SD1403 was highly conserved, and the homology of nucleotide sequence with other reference strains was 94.8%–98.5%. From phylogenetic trees constructed on the basis of genomic sequences, the SD1403 and GD-K-1 were closely related, and results were very similar to the phylogenetic trees constructed on the basis of VP1, showing that VP1 is more representative between the strains.

It was reported that pathogenicity and infectivity of GD-K-12 were weak [3], so we speculated that SD1403 might also be an attenuated strain. Previous studies indicated that if AA 139 and/or AA 144 of VP1 were glutamines, the infectivity and replication were weak. Both AA 139 and 144 of VP1 from SD1403 were glutamines, and these traits were in accordance with GD-K-1 and AH6 strains which were isolated in China. We observed that the SPF chicken population might have been infected with the SD1403 strain for a long time, and the results found that it did not show obvious pathogenicity. It also revealed that SPF chickens raised at the same time did not have pathogenicity, indicating that the pathogenicity and transmissibility of SD1403 strain were weak. According to reports, vaccines contaminated with ALV and REV might cause more serious damage, maybe because ALV and REV have relatively strong pathogenicity [15]. The transmissibility and pathogenicity of SD1403 were weak without showing clinical signs and symptoms. Perhaps this was an important reason why chicken populations were infected with SD1403 latently. Maybe because ALV, REV, and CIAV have different pathogenicity, the avian vaccines contaminated by REV and ALV were more than those by CIAV in the past years.

Although the CIAV infection in SPF chicken was relatively weak, we could not ignore its potential damage. On the one hand, when vaccine contaminated with CIAV is used in a younger chicken population, the pathogenicity of CIAV might be enhanced; on the other hand, if some chicken populations are infected with both ALV and REV, the pathogenicity of CIAV could also be enhanced [16]. Therefore, we must keep detecting CIAV in SPF chicken populations and attenuated vaccines.

## Figures and Tables

**Table 1:** Information of CIAV reference strains and amino acids in highly variable regions of VP1 protein.

Strains	Country and time	Accession number	Whole length	Amino acid position of VP1	
				139#	144#
M81223	1993, Germany	M81223	2298 bp	Lysine	Aspartate
CAU65414	1996, Australia	CAU65414	2298 bp	Glutamine	Glutamine
TR20	1999, Japan	AB027470	2298 bp	Glutamine	Glutamine
L14767	1999, USA	L14767	2298 bp	Glutamine	Glutamine
A2	2000, Japan	AB031296	2298 bp	Lysine	Glutamine
AF227982	2001, Australia	AF227982	2286 bp	Lysine	Glutamine
AB046590	2001, Japan	AB046590	2298 bp	Lysine	Glutamine
AF475908	2002, China	AF475908	2298 bp	Lysine	Glutamine
clone 33	2002, Germany	AJ297684	2298 bp	Lysine	Glutamine
SMSC-1P60	2003, Malaysia	AF390102	2298 bp	Lysine	Glutamine
BD-3	2004, Bangladesh	AF395114	2298 bp	Glutamine	Glutamine
TJBD40	2004, China	AY846844	2298 bp	Lysine	Glutamine
AH6	2005, China	DQ124935	2298 bp	Glutamine	Glutamine
TJBD33	2005, China	AY843527	2298 bp	Lysine	Glutamine
SMSC-1P123WT	2005, Malaysia	DQ217401	2298 bp	Lysine	Glutamine
CAE26P4	2007, Netherlands	D10068	2298 bp	Lysine	Glutamine
3711	2007, Australia	EF683159	2279 bp	Lysine	Glutamine
C14	2007, China	EF176599	2298 bp	Lysine	Glutamine
01-4201	2007, USA	DQ991394	2298 bp	Lysine	Glutamine
SDLY08	2008, China	FJ172347	2298 bp	Lysine	Glutamine
CAECA123	2008, Japan	D31965	2319 bp	Lysine	Glutamine
AB119448	2009, Japna	AB119448	2298 bp	Glutamine	Glutamine
98D02152	2010, USA	AF311892	2298 bp	Lysine	Glutamine
GD-1-12	2012, China	JX260426	2298 bp	Lysine	Glutamine
GD-K-12	2013, China	KF224935	2298 bp	Glutamine	Glutamine
GD-L-12	2013, China	KF224936	2298 bp	Lysine	Glutamine
CIAV89-69	2013, Korea	JF507715	2298 bp	Lysine	Glutamine
CAV-10	2014, Argentina	KJ872513	2298 bp	Glutamine	Glutamine
SC-MS	2014, China	KM496305	2298 bp	Lysine	Glutamine
NC001427	2015, USA	NC001427	2319 bp	Lysine	Aspartate
SD1403	2016, China	KU221054	2298 bp	Glutamine	Glutamine

**Table 2:** Primers used for genome amplification.

Primers	Sequence	Location	Temperature	Product length
F1	5′-GCATTCCGAGTGGTTACTATTCC-3′	# 1–23	55°C	842 bp
R1	5′-CGTCTTGCCATCTTACAGTCTTAT-3′	# 819–842		

F2	5′-CGAGTACAGGGTAAGCGAGCTAAA-3′	# 743–767	55°C	990 bp
R2	5′-TGCTATTCATGCAGCGGACTT-3′	# 1712–1732		

F3	5′-ACGAGCAACAGTACCCTGCTAT-3′	# 1643–1664	55°C	802 bp
R3	5′-CTGTACATGCTCCACTCGTT-3′	# 87–151		
